# Pharmaceutical Residues in Human Urine from Patients Under Antiretroviral Regimens and Self-Reported Healthy Individuals Before and After Three Months’ Storage: Implication for Agricultural Reuse in Pretoria, South Africa

**DOI:** 10.3390/biotech15040092

**Published:** 2026-10-13

**Authors:** Mugivhisa Liziwe Lizbelt, Baloyi Khanyi Faith, Oluwaseun Mary Oladeji, Olatunde Stephen Olatunji, Joshua Oluwole Olowoyo

**Affiliations:** 1 Department of Biology and Environmental Sciences, Sefako Makgatho Health Sciences University, Pretoria 0204, South Africa; liziwe.mugivhisa@smu.ac.za (M.L.L.); khanyibaloyi7@gmail.com (B.K.F.); 2 Discipline of Chemistry, School of Agriculture and Science, College of Agriculture Engineering and Science, University of KwaZulu-Natal, Westville, Durban 4000, South Africa; olatunjio@ukzn.ac.za; 3 Department of Health Sciences and The Water School, Florida Gulf Coast University, Fort Myers, FL 33965, USA

**Keywords:** pharmaceuticals, LC-MS, metformin, urine storage, antiretroviral

## Abstract

**Background/Objectives**: Pharmaceutical residues in human urine have raised serious concerns about the safe reuse of urine in agriculture. The current study investigates and compares the concentrations of pharmaceuticals in urine samples collected from male patients undergoing active antiretroviral regimens and self-reported “healthy individuals” at a government hospital in Pretoria, South Africa, and determines how the residues changed during three months of ambient storage, which is the usual recommendation before use in agriculture. **Methods**: The urine samples were collected in a plastic bottle to prevent fecal contamination, and 15 targeted compounds were analyzed using solid-phase extraction and liquid chromatography–mass spectrometry. The linearity ranged from 0.9910 to 0.9999, 75.5% to 99.1% for recovery, and intra-day and inter-day precision were below 6%. The urine from each group was collected into one pooled sample and then analyzed both pre- and post-storage. **Results**: Thirteen pharmaceuticals were detected in urine from patients on regular medication before storage; in “healthy individuals”, detections rose from eight to twelve after storage. Concentrations in the composite samples after storage were higher for some of the compounds but lower for others, including caffeine, nevirapine, prednisolone and 17α-ethinylestradiol. Metformin was highest overall at 1186.25 µg/L and remained at 806.68 µg/L after storage. The highest concentration for all the pharmaceuticals was reported for metformin, with a value of 1186.25 µg/L, occurring in the group of patients on a regular regimen. There could be a possibility that the rise can be attributed to the hydrolysis of conjugated metabolites during storage; however, no analysis of conjugates was carried out. **Conclusions**: This study shows the need for appropriate treatment of human urine prior to its reuse as fertilizer and echoes the importance of further research into human exposure pathways and effective pharmaceutical removal strategies.

## 1. Introduction

The use of human urine as organic fertilizer has been documented widely in the literature because of the presence of valuable plant nutrients, such as nitrogen, phosphorus and potassium [1]. Inorganic fertilizers such as NPK have been widely used for agricultural purposes to increase crop yield and provide food for the ever-increasing world population. However, excessive use of chemical fertilizers to increase crop yield has been associated not only with a decline in soil organic matter that contributes to decreased crop production but also with pollution to the immediate environment [2,3], including water contamination and soil nutrient loss [4]. Hence, there has been a need to find more sustainable organic fertilizers, such as human urine, plant or animal manure, and biosolids, to produce food through agriculture.

Organic fertilizers such as human urine have been considered as potential alternatives to chemical fertilizers [5]. Studies conducted by Akpan-Idiok et al. [6] showed that all the organic fertilizers used in their studies for the fertilization of various crops, including human urine, yielded a better outcome because these contain important macronutrients, such as nitrogen in the form of urea, potassium, and phosphorus. In addition, compared to chemical fertilizers, human urine is easy to apply, and it contains water and different inorganic compounds such as uric acid, creatinine, and organic compounds like chloride, phosphate, and ammonia [1]. In addition, human urine is freely available in all human societies, making it affordable at no cost to even poor farming communities compared to expensive chemical fertilizers [7]. In Germany, human urine has been shown to have a higher fertilizing effect than mineral fertilizers in barley production [8]. In Ethiopia, the yields of Swiss chard were four times higher when fertilized with human urine than those that were not fertilized [8]. In Pretoria, South Africa, Mugivhisa and Olowoyo [9] demonstrated a positive influence in the yield of *Spinacea oleracea* harvested from soil to which urine was added.

Studies have, however, also reported that human urine may contain some toxic chemicals and other compounds depending on the source, collection, storage methods, the content of the human urine, and mode of application, among other factors [10]. For instance, the study of Mugivhisa and Olowoyo [9] showed that human urine may contain certain heavy metals that can later be taken up and bioaccumulated by plants. The study of Gao et al. [11] also reported that the concentrations of some pharmaceuticals in the excreted human urine were higher than the required amounts. Deeks et al. [12] also noted that there is a partial transformation in the degradation of pharmaceuticals in the human body, and most of the pharmaceuticals excreted through urine and feces are released in their original form as metabolites. Therefore, using human urine in organic farming has a potential risk of releasing pollutants such as pharmaceutical residues into the environment and cultivated plants [13].

Pharmaceuticals serve a crucial role in maintaining today’s modern life [14]. However, due to the extensive use of pharmaceuticals, there are undesired worldwide problems with environmental pollution caused by the introduction or disposal of used and unused pharmaceuticals into the aquatic and terrestrial environments [15,16]. Pharmaceutical residues have received a lot of attention as Emerging Organic Contaminants (EOCs) in the environment in the last three decades [17]. Some adverse effects caused by pharmaceuticals in the environment include sterility, feminization of aquatic animals, bacterial resistance to antibiotics and other drug resistance of pathogenic organisms [18]. Therefore, the environmental fate of pharmaceuticals excreted through urine needs to be investigated to prevent the public from being exposed to pharmaceuticals that are excreted with human urine and feces which are also used as soil amendments in the cultivation of food crops [19].

Once the pharmaceutical is excreted as metabolites in urine, it can undergo biodegradation in the environment [20]. However, some pharmaceuticals do not easily undergo biodegradation in the environment, resulting in them becoming persistent [20,21]. Modern technologies have made it possible to track pharmaceuticals after they have been excreted from the body. Studies have shown that pharmaceutical contaminants can be found in urine, sewage, surface water and soil [22,23,24,25]. Environmental persistence, biological toxicity and bioaccumulation of pharmaceuticals may cause potential hazards and risks to the environment and human health [22]. It has been established that a small portion of pharmaceuticals are absorbed into the human body and most of the original pharmaceuticals are excreted as metabolites in urine [7,26]. Some of the pharmaceutical residues excreted through human urine may include antiretroviral drugs (ARVs) such as nevirapine, which are used to treat retroviral infections, among others [27]. Therefore, if urine is used to fertilize soil for crop production and fodder plants outside of the households, then it needs to be stored for at least one month [28]. Despite its relevance in agriculture, the use of human urine requires continuous probing into its content and impact on agriculture.

Stintzing et al. [29] and the World Health Organization [30] have suggested storage over a period of 30–60 days to allow for urea hydrolysis, which raises urine pH and results in the production of ammonia. However, there is limited information on the storage of urine, and the levels of pharmaceuticals present in them. Available studies have indicated the impact of storage period and temperature on the availability of pharmaceuticals in urine and this could either increase the concentration or decrease it, depending on the metabolites and microorganism present in the urine during storage [31,32]. Research has also suggested that concentrations of pharmaceutical residues in urine may not reach the point at which plants are negatively affected [22]. The urine that is meant for fertilizing any kind of crop must be kept for six months at 20 °C or above to be deemed hygienically safe against pathogens and viruses [33,34]. Kirchmann and Pettersson [33] indicated that human urine collected in a hygienic system can be used directly as a liquid fertilizer. However, the debate as to whether shorter or longer storage without any treatment can reduce pharmaceuticals is still ongoing and requires further investigation. However, according to Jaatinen et al. [35], storage is not sufficient to remove pharmaceutical residues in urine. Okem et al. [36] reported that education on the appropriate use and technical interventions and the development of appropriate guidelines on the use of urine should be developed and implemented by local authorities. From different studies, it is evident that the use of urine even with the levels of pharmaceuticals present in it may not lead to their accumulation in crop tissues at significant levels [37,38]. However, numerous studies have been done on crops using either treated sewage water, biosolids or manure fertilizers, where some pharmaceuticals have been shown to be absorbed through the roots and translocated into the edible plant parts with huge differences in levels between chemicals and species [39,40]. Studies have also suggested that fresh urine collected from healthy individuals may contain concentrations of pharmaceuticals ranging from low to high [41]. The use of urine from family members without storage is still common and widespread in some developing countries in Africa, as it is commonly believed that urine from a supposedly healthy individual may not contain pollutants, including pharmaceutical residues, that may affect the product [42]. The present study therefore seeks to determine the pharmaceutical contents of human urine collected from hospital patients on a medication regimen and “healthy individuals” (not on reported medication) stored for a period of three months prior to its application as organic fertilizer in Pretoria, South Africa. It is envisaged that the current research will contribute to a better understanding of the safety of urine-derived fertilizers and support the development of guidelines for their sustainable use in agriculture in Pretoria, South Africa. It should also be noted that this study was carried out in Pretoria, South Africa, a region characterized by warm temperature with direct influence on pH and microbial activities, which may affect the degradation of pharmaceutical compounds during storage [43,44].

## 2. Materials and Methods

Only males were approached for urine collection to avoid the possible interference of hormones and urinary tract infections (UTIs) that are common in female urine samples [45]. For the control group, “healthy male participants” from the university who reported that they were not on any medication were approached for urine collection. The purpose of this study was fully explained to all the participants. Consent from each participant to take part in this study was first sought and each participant had to sign a consent form upon agreement to donate urine samples. The patients were chosen because they followed a strict medication regimen (Table 1). The urine samples were collected into sampling plastic bottles to avoid any mixture with feces and stored separately in the freezer in the laboratory until there was enough urine to commence this study. The freezing of the urine was carried out to preserve the pharmaceuticals in the urine until enough urine was collected. When there was enough urine, it was then defrosted and stored at room temperature in a sealed container for a period of 3 months before analysis. Urine collected from “healthy individuals” was stored the same way before analysis. Patients from the clinic followed a regular medication regimen of different pharmaceuticals.

The regimen details were collected from the hospital participants themselves and consisted of antiretrovirals only. Other medication used by the participants for their comorbidities, over-the-counter drugs, or supplements taken from food were not accounted for, nor was any medication history taken from the unmedicated participants.

Chemical analysis:

Extraction and quantification of pharmaceuticals in collected urine samples:

Solid-phase extraction of the urine: Extraction was done using the Strata-X-C 33 µm polymeric strong cation cartridges (200 mg/6 mL SPE tubes) under vacuum. The cartridges were conditioned with 5.0 mL milliQ water, 7.0 mL MeOH and 7.0 mL milliQ water. Ten milliliters (10 mL) of each urine sample were then loaded onto the cartridges and allowed to elute under gravity. The cartridges were then rinsed with 1 mL Milli-Q water, and the water was discarded as waste. The cartridges were then washed in 2 cycles of 3 mL MeOH to recover the retained pharmaceuticals, making a total of 6.0 mL. The recovered eluate analytes were concentrated under a gentle air stream to near dryness and then reconstituted with 1.0 mL MeOH. The sample clean-up involved the use of cartridges of 0.5 mg silica gel, weighed and stacked in empty SPE glass tubes plugged with membranes to prevent the loss of the silica gel. The silica gel cartridge was then conditioned with 6.0 mL MeOH + acetone (3:1) and left to drain out as waste. The reconstituted samples in 1.0 mL MeOH were then loaded into the cartridge one at a time and collected. The silica gel was changed, and the SPE and membranes were washed with Milli-Q water between each sample to avoid cross contamination. The samples were then filtered into liquid chromatography (LC) vials using nylon syringe filters (diameter: 25.0 mm, pore size: 0.22 µm) for liquid chromatography–mass spectrometry (LC-MS) analysis.

Instrumentation and quantification: Pharmaceuticals in the urine were quantified using a liquid chromatograph coupled to a photodiode array detector and mass spectrometer (LCMS–2020) (Table 2). The method of Castiglioni et al. [46], Archer [47], and Inarmal and Moodley [48] was adapted but with slight modifications. The instrument was optimized and set at a flow rate of 0.22 mL/min for a sample injection volume of 5.00 µL. Separation was achieved via gradient elution using ultra-pure Millipore water modified by buffering with 5 mM ammonium acetate and 3 mM acetic acid as solvent A, and absolute methanol (MeOH) as solvent B as mobile phase (gradient programmed in Table 3), with a Shimadzu, Shim-pack GIST Reverse Phase C18 column with specification dimensions: 2.1 × 100 mm, 3 µm. Upon analysis, the concentrations of the pharmaceuticals and metabolites present in the samples were obtained by interpolating the peak area from the signal into the equation of the best fit deduced from the standard calibration curve.

Validation of the analysis was done based on the ICH Q2(R2) guideline, including the following parameters: linearity, detection and quantification limits, accuracy and precision. Calculation of LoD and LoQ was performed using a formula (3.3σ/S and 10σ/S, where σ is the standard deviation of the response and S is the slope of the calibration curve). Accuracy of the analysis was expressed in terms of recovery from urine samples spiked at three concentrations of analyte (5, 50 & 500 µg/L). Intra-day and inter-day precisions were evaluated using RSD calculation measurements performed within one day and across three different days, respectively.

### Data Analysis

Urine samples from each group were pooled together into a composite sample before extraction, and each composite sample was analyzed as a single observation for each analyte per time point. This does not allow for replication of data within each group, and thus no inferential statistical analysis was conducted. Concentration values are thus reported as single measurements, and any comparison made between groups or across different time points is purely descriptive. Differences noted in this paper should thus be understood as differences between composite samples and not as statistically significant effects. The percentage of change during the storage was determined as follows:

$$\frac{C_{Before}-C_{After}}{C_{Before}}$$

where C_before_ and C_after_ are the concentrations of each analyte measured before and after the three months of storage, respectively; in cases where the analyte concentration was below the limit of detection, the percentage decrease is presented as the minimal percentage calculated from the limit of detection.

## 3. Results

The results for method development and validation are presented in Table 4. This includes coefficients of determination, limit of detection, limit of quantification, recovery and inter-day and intra-day precision. The method had a proportional response to increased concentrations, with linearity ranging from 0.9910 to 0.9999 from metformin (y = 98,799x + 107,870) to lamivudine (y = 215,943x + 45.1). The LoD ranged from 0.009 µg L^−1^ (ciprofloxacin) to 0.842 µg L^−1^ (prednisolone), and the LoQ from 0.027 µg L^−1^ (ciprofloxacin) to 2.526 µg L^−1^ (prednisolone). The analyte accuracy (recovery) observed for the tested pharmaceuticals ranged from 75.54% for sulfamethoxazole to 99.10% for EE2; all compounds fell within the 80–120% range, except for sulfamethoxazole (75.54%) and acetaminophen (77.35%). The intra-day and inter-day precision of the method were all below 6% for all tested pharmaceuticals (Table 4).

### Pharmaceuticals in Urine

**Figure 1:**
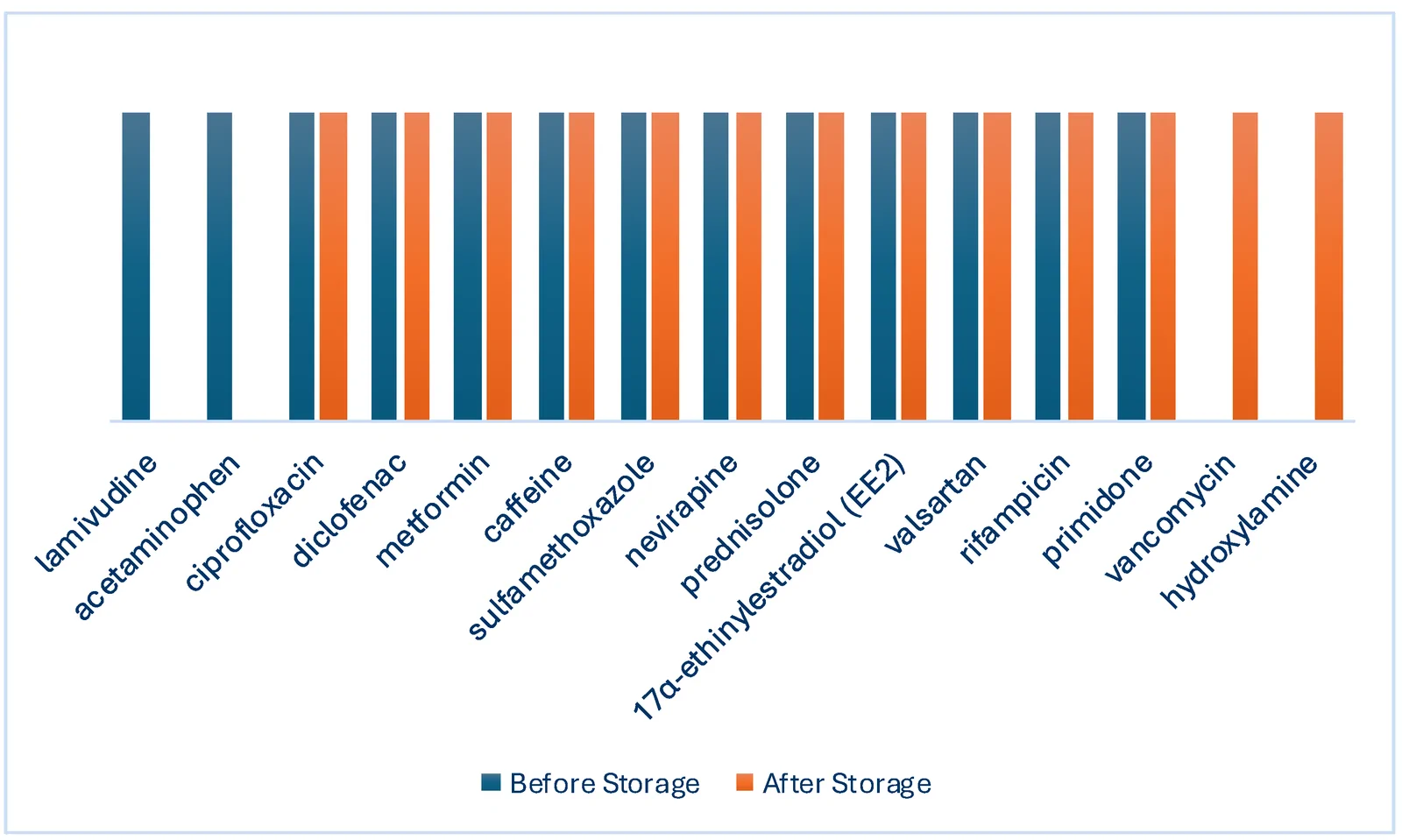
Pharmaceuticals detected in the urine of hospital patients before and after three months storage.

**Figure 2:**
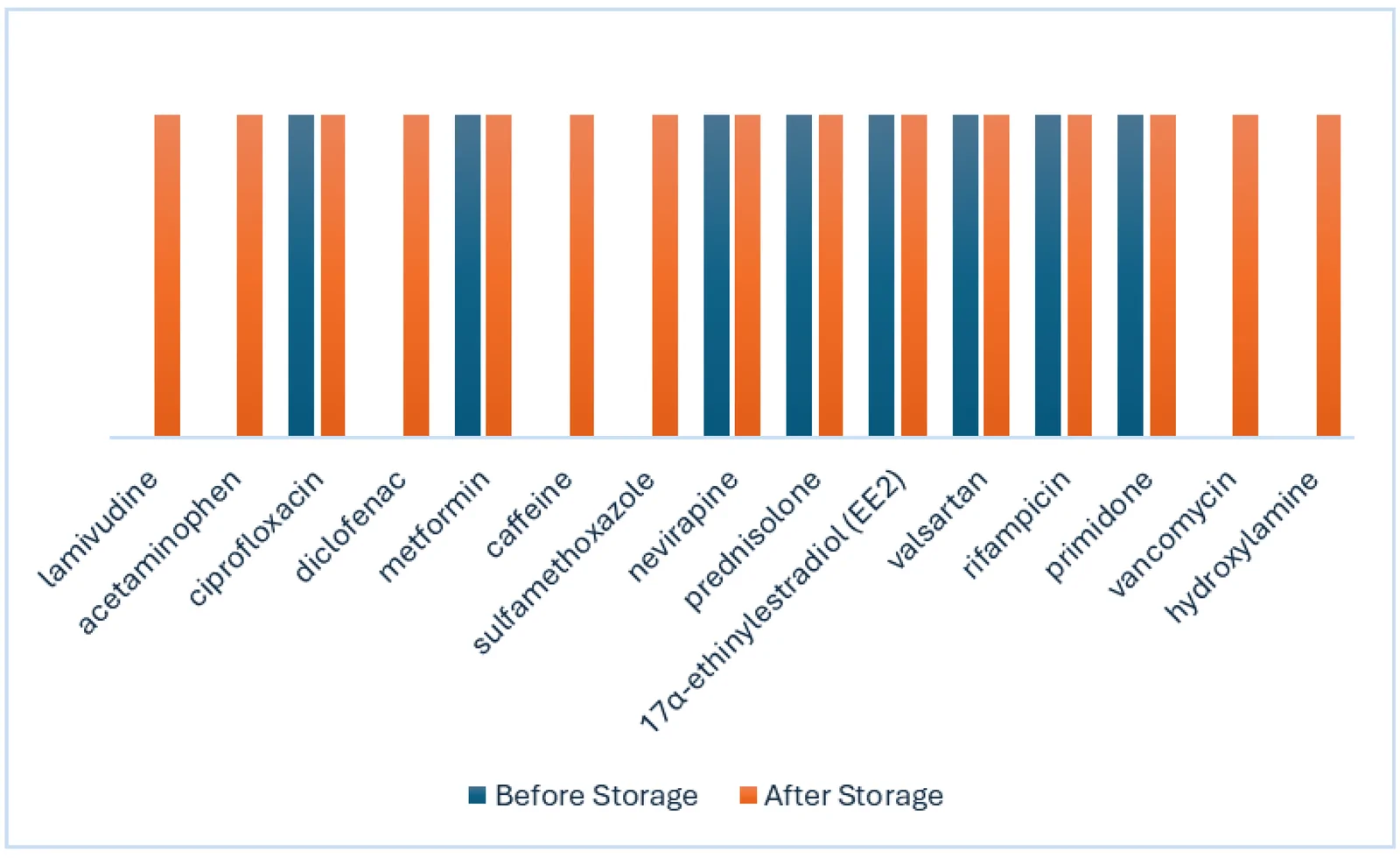
Pharmaceuticals detected in the urine of individuals not on a regular regimen (control) before and after three months storage.

The pharmaceuticals detected in urine samples collected from hospital patients and the “healthy individuals” urine samples before and after storage are depicted in Figure 1 and Figure 2, respectively. The pharmaceuticals found in urine samples from hospital patients before storage were lamivudine, acetaminophen, ciprofloxacin, diclofenac, metformin, caffeine, sulfamethoxazole, nevirapine, prednisolone, 17α-ethinylestradiol (EE2), valsartan, rifampicin, and primidone (Figure 1), and after three months of storage, the pharmaceuticals detected in the urine samples collected from hospital patients were vancomycin, ciprofloxacin, diclofenac, metformin, caffeine, sulfamethoxazole hydroxylamine, nevirapine, prednisolone, 17α-ethinylestradiol (EE2), valsartan, rifampicin, primidone, and sulfamethoxazole hydroxylamine (Figure 1). Lamivudine and acetaminophen were observed to be present in the urine before storage but absent from the results obtained in the urine after storage (Figure 1). It was noted that two pharmaceuticals, vancomycin and hydroxylamine, which were not present at pre-storage, were observed after storage for three months, while lamivudine and acetaminophen were not detectable.

**Figure 3:**
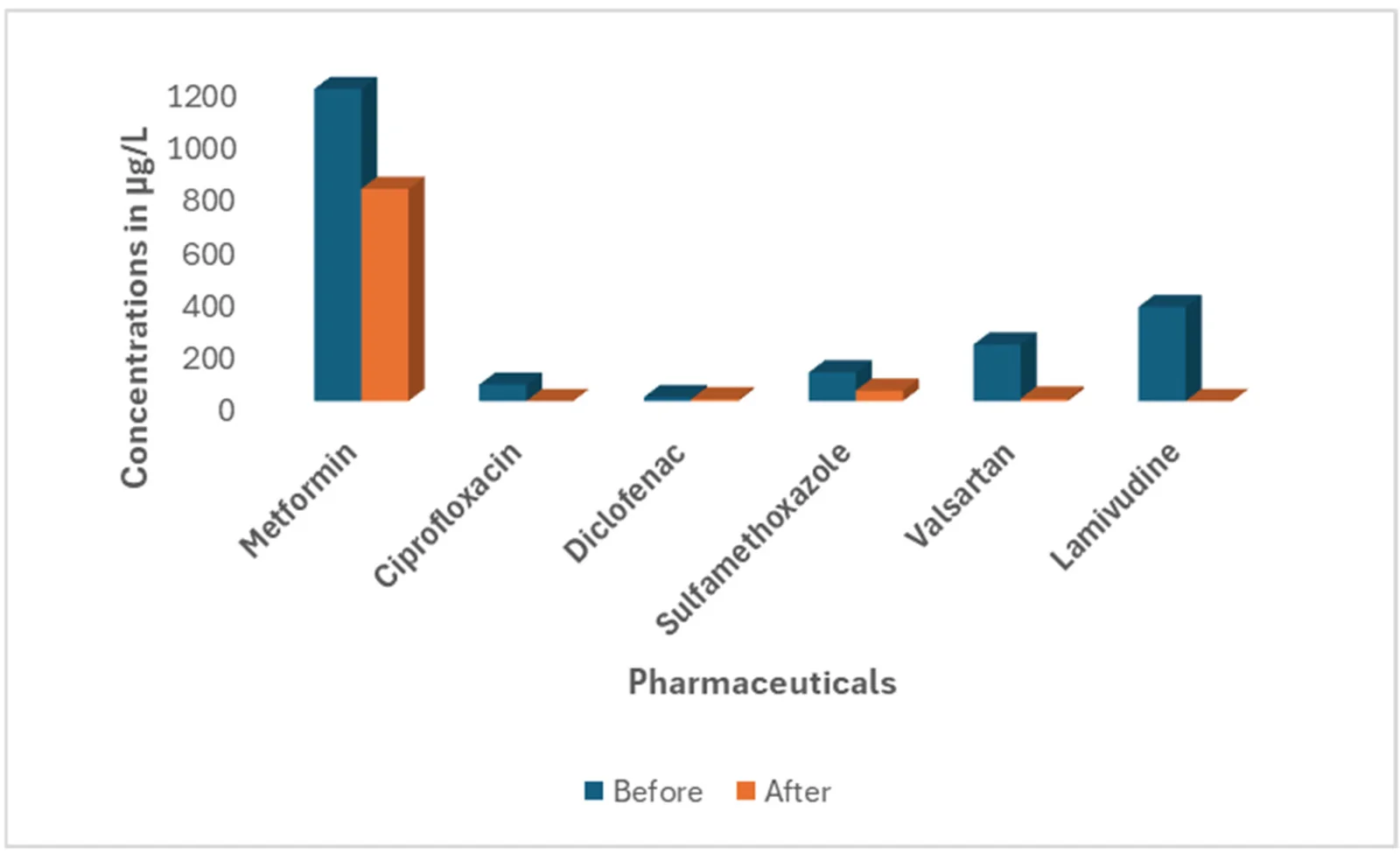
Pharmaceuticals with a decrease in concentrations after storage for 3 months.

The concentrations of pharmaceuticals detected in the post-storage composite samples were lower compared to the pre-storage composite sample. Metformin was reduced by 32%, ciprofloxacin and lamivudine did not occur at detectable levels (probably 100% decomposed), diclofenac reduced by 51.4%, sulfamethoxazole by 63.8%, and valsartan by 95.4% (Figure 3). There were no forms of treatment for the above except that the samples were stored under room temperature for a period of three months for the urine samples of the “healthy individuals”. In the unmedicated group, only four out of the fifteen tested pharmaceuticals decreased in concentrations: namely, ciprofloxacin from 10.62 µg/L to 7.55 µg/L, nevirapine 47.85 µg/L–22.2 µg/L, 17α-ethinylestradiol (EE2) from 23.87 µg L^−1^ to 19.91 µg L^−1^, and primidone from 123.18 µg L^−1^ to below detection limit.

The results also showed an increase in the concentrations of some pharmaceuticals in the two groups. For instance, caffeine increased from 61.89 µg L^−1^ to 155.74 µg L^−1^ in the group on a regular medication regimen, while it was not detected in the “healthy individuals” group prior and after storage (Table 5).

Six of the pharmaceuticals were not detected in the urine samples collected from the “healthy individuals” group before the storage period but were later reported to be above the detection limit after a period of three months (Table 5). Sulfamethoxazole hydroxylamine was the only analyte that displayed consistency in the direction of its variation in both samples. In the patient samples, sulfamethoxazole hydroxylamine increased from below detection level to 40.33 µg L^−1^, while in the unmedicated samples, it increased from below detection to 22.0 µg L^−1^. Nevirapine, 17α-ethinylestradiol and primidone increased in concentration in the urine after storage but decreased in concentration in the “healthy individuals” group.

In all, the highest concentration for all pharmaceuticals was reported for metformin, with a value of 1186.25 µg/L occurring in the group of patients on a regular regimen. The observed levels may be attributed to the endogenous concentration of the pharmaceutical substances and their metabolites following the regimen. The concentration of metformin was 806.68 µg/L even after storage. The highest concentration of pharmaceuticals recorded from the “healthy individuals” group was reported for lamivudine, with a value of 116.92 µg/L. The concentrations in the composite samples from healthy individuals without regular medication were relatively low compared to those in the composite samples of individuals taking regular medication.

## 4. Discussion

The results from the current study revealed that of the fifteen pharmaceuticals investigated, thirteen pharmaceuticals were found to be present in the urine samples from hospital patients before storage and eight pharmaceuticals were found in the urine samples from “healthy participants” before storage. The results showed that pharmaceuticals are indeed excreted in urine. Lienert et al. [49] conducted a meta-analysis on excretion pathways of 212 pharmaceuticals and found that the majority (64%) of each metabolized pharmaceutical was excreted through urine, while 35% was excreted with feces [49]. In the current study, all participants were male patients and were provided with an appropriate collection bottle that will not allow for a mixture of urine and feces; so, the presence of pharmaceuticals could only be linked to the metabolites found in the urine. Ozel Duygan et al. [50] also found the presence of pharmaceuticals from source-separated urine, including diclofenac (11–14 µg L^−1^), hydrochlorothiazide (66–110 µg L^−1^) and atenolol acid (4–14 µg L^−1^). Pharmaceuticals found in human urine have a longer half-life and more complex structures, which make them take longer to degrade [7]. The presence of pharmaceuticals in source-separated urine used as fertilizers is of great concern as this could lead to pharmaceuticals entering the food chain after plant uptake and cause a potential risk to animal and human health [51].

The urine in the current study was stored for three months, and it retained a total number of 13 pharmaceuticals in the urine samples from hospital patients out of the 15 pharmaceuticals that were investigated; however, some of the pharmaceuticals reported were not identical to those reported in the sample before storage. Some of the pharmaceuticals, such as metformin, ciprofloxacin, diclofenac, sulfamethoxazole, and valsartan, decreased in concentration after storage for three months. These reductions during storage do not seem likely to be due to volatilization since the vapor pressure and Henry’s Law constant for the compounds of interest are relatively low. These reductions are more likely caused by microbial metabolism due to the rapid growth of urease-producing microorganisms, which grow at room temperature (24 °C in this study). Hydrolysis and instability of the compounds under the alkaline (pH∼9) and ammonia-enriched environment created via hydrolysis of urea are also factors, as is adsorption onto precipitated materials like struvite and calcium phosphates [50,52]. Such metabolic pathways can also result in an elevation in the concentrations of some parent compounds via microbial deconjugation of glucuronides, sulfates, and acetyl compounds. The authors found several prescribed pharmaceuticals and/or their metabolites (e.g., sulfamethoxazole, SMX-hydroxylamine, lamivudine) in the stored urine samples of non-treated patients.

Studies on the degradation of pharmaceuticals during urine storage are still very scarce; however, a study by Gajurel et al. [53] showed that diclofenac was not removed from urine even after a year of storage and sulfamethoxazole decreased by 30% in a year or by 24% during 6 months of urine storage [54]. In the current study, metformin was reduced by 32%, ciprofloxacin was not detected (100%), diclofenac was reduced by 51.4%, sulfamethoxazole by 63.8%, valsartan by 95.4% and lamivudine was not detected. Winker et al. [41], reported that even after 1 and a half years of urine storage, some beta-blockers and antibiotics were still present [50].

The urine from “healthy participants” samples had more pharmaceuticals after three months of storage. A study by Ozel Duygan et al. [50] is consistent with the findings of the current study, which found that some pharmaceuticals increase during storage. The study further reported that the concentration of sulfamethoxazole, the parent of N4-acetylsulfamethoxazole, increased, which suggests transformation of N4-acetylsulfamethoxazole into sulfamethoxazole during storage. According to Chow [55], two pharmaceutical products may contain the same active particles, and they could be considered bioequivalent or pharmaceutical alternatives of one another. Therefore, bioequivalence studies are required to determine if the pharmaceuticals identified after storage were equivalent to those identified before storage by NIST matching; however, that was not in the scope of the current study [55]. Nara et al. [56] also reported that phenethylamine was not detected in urine samples stored at −20 °C throughout the 14-day period. Nevertheless, phenethylamine was detected after 6 days in these samples stored at 4 °C and after only 1 day in samples stored at 22 °C. Additionally, the concentration of phenethylamine in these samples increased each day after detection.

The urine samples that were collected from participants who were “healthy” and not on any self-reported pharmaceuticals had a lower variety of pharmaceuticals as compared to the samples collected from hospital patients on medication. In all, the pharmaceuticals found in urine had a higher concentration compared to those that have been found in water bodies in South Africa. Madikizela et al. [57] investigated the presence of pharmaceuticals and their transformation products in Klip River (South Africa) and found that 47 pharmaceutical compounds out of 92 that were screened were present in the river, 31 of which were detected for the first time in South Africa. Pharmaceuticals such as acetaminophen (0.432 µg L^−1^), oxolinic acid (0.355 µg L^−1^) and flumequine (0.432 µg L^−1^) were detected. The current study identified the presence of acetaminophen in the urine of “healthy participants” after storage of three months to be 62.28 µg L^−1^. According to Madikizela et al. [57], the results from the literature indicated that three months of storage was not enough to substantially degrade or remove pharmaceuticals. Other studies even suggested that the urine of people who are on any medication should not be used as fertilizers in agriculture [58,59].

The World Health Organization (WHO) recommends a urine storage time between 1 and 6 months depending on the crops and temperature at which the urine will be applied [30]. Large-scale storage of collected urine requires a storage period of at least three months or longer for proper hygiene to be achieved [60]. The current study stored the urine at a room temperature for a period of about three months following a common practice in the area. This was done with the aim of providing needed information for the public on the need for proper storage. Laparre et al. [61] reported that storage conditions, such as the temperature, may affect the stability of the urine as the study concluded that urine samples stored at −20 °C exhibit a global stability over a long period with no major modifications compared to that stored at the −80 °C condition. The current study noted a decrease in some pharmaceuticals and an increase in some pharmaceuticals after storage; hence, studies are needed to understand the behaviors of each of these pharmaceuticals for proper application in agriculture, especially from peasant farmers.

The current study relied on self-reported information from “healthy individuals” that are not on a regular medication regimen. From the findings of this study, the “healthy individuals” urine contained some pharmaceuticals that were also recorded for patients on an active regimen. For instance, an antiretroviral drug called lamivudine was detected in the composite samples from both sets following storage. No information regarding medication was recorded for the healthy-sample set, and the composite may consist mostly of a few donors, meaning that it will be impossible to identify the origin of this substance based on the current data set. Some possibilities might include prescribed or prophylactic use of the substance, and some type of interference. Antiretrovirals have been found as ingredients of the street drug nyaope [62], suggesting further investigation into the source of these pharmaceuticals in the urine of health individuals, which may also lead to stricter measures on the distribution of these antiretroviral drugs. This suggests that reliance on self-reported health to use urine samples from individuals may not be a good method for the determination of safe urine for agricultural purposes. Urine from healthy individuals may contain pharmaceuticals and their metabolites, which may come from prescribed drugs, over-the-counter use, diet, and environmental exposure, as noted in our current study. A similar assertion was made by Bischel et al. [51] on different types of pharmaceuticals in human urine intended for fertilizer production. However, it should be noted that the levels of pharmaceuticals obtained from the “healthy participants” group are very low when compared with those on active medication. This is consistent with the finding that the concentrations of these pharmaceuticals are usually low but they may be present at levels far above those of environmental waters, making it both a useful biomonitoring matrix and a focus for environmental risk management [22].

Several pharmaceuticals and metabolites such as acetaminophen, diclofenac, sulfamethoxazole, sulfamethoxazole hydroxylamine, and lamivudine were found in the urine of individuals who declared themselves healthy after storage in the urine. Their concentrations (in tens of µg/L) are orders of magnitude higher than those recorded for South African surface waters [57] and thus cannot be explained by contamination from drinking water or foods and indicate usage of these pharmaceuticals. Acetaminophen, diclofenac, and caffeine are all commonly purchased without a prescription or are contained in drinks. Sulfamethoxazole is used predominantly in South Africa as part of the combination preparation co-trimoxazole and is used in prophylaxis in HIV-positive people, while the detection of lamivudine can be an indication of unreported antiretroviral or prophylactic treatment use [62]. One of the reasons why these substances are detected only when stored may be the release of parent compounds from their conjugated metabolites in the body. Acetaminophen is primarily excreted as glucuronide and sulfate conjugates, diclofenac as a readily hydrolysable acyl glucuronide, and sulfamethoxazole as N4-acetyl-sulfamethoxazole that can undergo deacetylation to form the parent compound during urine storage [50]. The process of microbial deconjugation and alkali hydrolysis of the stored urine (pH ≈ 9) could potentially increase the levels of the parent compound above their respective LODs. Conjugated metabolites, pH of urine, and microbial activity were not measured during storage and hence this is a possible mechanism. However, lamivudine is primarily excreted as the unmetabolized parent compound; so, the reduced matrix effect due to the hydrolysis of urea may have also influenced the increased concentration of this compound. The volume of the samples was not determined before and after the storage process, and therefore, evaporation cannot be ruled out or even accounted for. Evaporation would have increased the concentration of all analytes to the same degree and therefore cannot account for the decrease in some and increase in other compounds.

The significance of the residue concentrations detected in this study will depend on the presence of these residues in the soil and their uptake into the plants. Their uptake will be dependent on speciation at soil pH, log Kow/log Dow, molecular weight and sorption in the soil. Neutral, moderately polar and low-molecular-weight compounds will be easily taken up and transported in the xylem to the leaves and fruits, while anionic compounds will be repelled by the negatively charged roots [63]. Out of the substances that accumulated or increased in concentration in this study due to storage, caffeine and primidone are neither positive nor negative and mobile. Primidone has a similar behavior to that of carbamazepine, which belongs to the group of drugs that is most often found in edible tissue of crops irrigated with treated wastewater [64]. Metformin, which has the highest concentration after storage (806.68 µg/L), has been demonstrated to have uptake and translocation in agricultural plants despite being highly polar, except that it is converted to guanylurea in soil [65]. However, diclofenac, valsartan, and sulfamethoxazole have been found to be predominantly anionic and will translocate less, while ciprofloxacin is highly adsorbed and accumulates mostly in plant roots [66]. 17α-ethinylestradiol is hydrophobic and largely sorbed to soil and roots, and rifampicin and ivermectin (820–875 Da) are not likely to be absorbed at a considerable extent. Data on the absorption of anti-retroviral drugs by plants, such as lamivudine and nevirapine, are extremely rare; this is a critical gap in the current knowledge for South Africa, where anti-retroviral drugs are widely used. Those compounds, which are persistent or accumulate upon storage, along with the parent compounds generated by de-conjugation, are the ones of significance when considering field loading, as parent compounds are usually more accessible to plants than conjugated compounds. These results provide an evidence base for the treatment of urine and further uptake experiments using fertilization with urine in crops.

Vancomycin and sulfamethoxazole hydroxylamine were not detected before storage but were detected after storage in both composite samples (40.33 and 22.0 µg/L). Sulfamethoxazole hydroxylamine is a stable metabolic derivative of the degradation of sulfamethoxazole. This probably accounts for its detection in the samples after storage, suggesting that sulfamethoxazole degraded during storage. In this case, the metabolization of sulfamethoxazole resulted in a 63.8% reduction in sulfamethoxazole concentration in the patient’s urine.

Limitations of this study: The first limitation in this study is that a single pooled sample from each group was used at each time point without replication. Secondly, the volume of the samples was not determined before and after storage and therefore the role played by evaporation in the concentration levels cannot be determined. Lastly, the conjugates and the pH were not determined and therefore the mechanisms put forward for the observed changes are hypotheses. Future studies can address these limitations by analyzing single or replicate pooled samples and determining their mass or volume at each time point.

## 5. Conclusions

The current study compared the presence of pharmaceuticals in the urine of patients on an active drug regimen in hospital with self-reported “healthy individuals” not on a regular regimen at a government hospital in Pretoria, South Africa, and analyzed how urine composition changed over three months of storage under ambient condition. This study screened fifteen different pharmaceutical analytes; only thirteen pharmaceuticals were detected in the urine of patients on regular medications. It should be noted from this study that from the urine of “healthy individuals”, eight out of the reported fifteen pharmaceuticals were found, and after three months of storage, 12 out of the 15 pharmaceuticals were found present in the samples. The presence of lamivudine in the urine of self-reported individuals is noteworthy, suggesting undisclosed medication usage or potential environmental or indirect exposure pathways requiring further investigation into the source of this medication in healthy individuals. This will assist policymakers, especially in the provision of adequate or stricter measures in the distribution of antiretroviral regimen drugs, as this has become a component of nyaope, a common cocktail used in the study area. In contrast with the unmedicated patients, the number of substances found increased rather than decreased, as acetaminophen, lamivudine, and sulfamethoxazole became detectable after storage. This result may point to microbial deconjugation of glucuronide and sulfate metabolites, resulting in parent compounds, and showed that storage alone may not offer adequate removal for certain pharmaceuticals in urine, as noted in this study. Moreover, the pharmaceutical profiles found in the patient urine samples were wider than the antiretroviral regimen medications in use would suggest, with traces identified even among those patients who had not taken any form of medication. Hence, there is no guarantee that either the prescriptions given to the individuals or their own assessment of their health condition will be enough to establish whether urine poses no risks to agricultural practice or not; chemical testing is necessary. Generally, based on the findings of this study, it is recommended that human urine undergoes an appropriate treatment method before its application as fertilizer to prevent pharmaceutical contamination and further study of the origin of the traces found in the unmedicated group will have to be conducted. There is also a need for further investigation on human exposure pathways to pharmaceuticals, probably in their diet (water and food), and effective ways to treat urine for the removal of pharmaceuticals.

## Figures and Tables

**Table 1:** Urine samples collected based on the different types of regimens and combination treatments of the patients.

Category	Regimen	Combination Treatment
1	Aluvia	Ritonavir/Lopinavir
2	Tribuss/Atripla	Efavirenz, Emtricitabine and Tenofovir
3	Volutrip	Dolutegravir, Lamivudine, Tenofovir
4	Cagol	Atazanavir (ATV), Lamivudine(3TC), Dolutegravir (DTG)/Abacavir
5	Triumeq	Abacavir, Lamivudine and Dolutegravir
6	Dumiva	Abacavir (ABC) and Lamivudine(3TC)

**Table 2:** Molecular ion peaks of the investigated pharmaceuticals analyzed using LC-ESI-MS (SIM mode).

Pharmaceuticals	Molecular Ions (*m*/*z*)	Transition Ions (*m*/*z*)	Mode
Sulfamethoxazole	254	156, 92, 94	[M + H]^+^
Sulfamethoxazole hydroxylamine	270	156, 99, 108/92	[M + H]^+^
Nevirapine	267	226, 198, 225, 107	[M + H]^+^
Rifampicin	823	791, 163, 107	[M + H]^+^
Metformin	130	60, 71	[M + H]^+^
Valsartan	436	391, 350, 304, 235, 192, 179, 116	[M + H]^+^
Ivermectin	877	874, 569, 551, 307,397	[M + H]^+^
17α-ethinylestradiol (EE2)	296	295, 144	[M − H]^−^
Caffeine	195	138, 110, 163, 151	[M + H]^+^
Prednisolone	361	147, 171, 343	[M + H]^+^
Lamivudine	230	112	[M + H]^+^
Acetaminophen	152	110, 114	[M + H]^+^
Ciprofloxacin	332	288, 231, 314	[M + H]^+^
Diclofenac	296	250, 214	[M + H]^+^
Primidone	219.2	162, 119, 91	[M + H]^+^

**Table 3:** Instrument parameters set up for the determination of the selected pharmaceutical and pharmaceuticals metabolites via gradient elution using LC-MS.

Hold Time (min)	Solvent A	Solvent B (MeOH)
0–1 min	80	20
1–18.5	80–10	20–90%
18.5–22.5	10	90% MeOH
22.5–25	10–80	90–20%
25–27.5	80	20%

**Table 4:** Method validation obtained from the analytes of interest.

Pharmaceuticals	R^2^	Equation	LoD µg/L	LoQ µg/L	Accuracy (Recovery%)	Inter-Day Precision	Intraday Precision
Sulfamethoxazole	0.9952	y = 36,054x + 7914.6	0.068	0.204	75.54	1.05	2.47
Sulfamethoxazole hydroxylamine	0.9965	y = 31,284x − 18,592	0.067	0.201	80.06	5.42	4.82
Nevirapine	0.9979	y = 76,202x + 17,370	0.063	0.189	98.50	2.04	2.74
Rifampicin	0.9940	y = 43,519x + 17,432	0.060	0.180	85.94	1.12	1.95
Metformin	0.9910	y = 98,799x + 107,870	0.306	0.918	90.27	4.20	3.80
Valsartan	0.9948	y = 30,975x + 9114.2	0.209	0.627	95.02	1.85	2.44
Ivermectin	0.9955	y = 41,490x + 26,166	0.545	1.635	85.98	1.40	1.98
17α-ethinylestradiol	0.9936	y = 13,320x + 1720	0.192	0.576	99.10	1.55	1.32
Caffeine	0.9941	y = 32,706x + 5734.7	0.028	0.084	92.59	2.89	2.63
Prednisolone	0.9972	y = 28,699x + 7707.8	0.842	2.526	97.00	2.41	2.94
Lamivudine	0.9999	y = 215,943x + 45.1	0.051	0.153	88.65	1.85	2.44
Acetaminophen	0.9997	y = 110,712x − 135.1	0.072	0.216	77.35	1.40	1.98
Ciprofloxacin	0.9987	y = 206,982x − 82.6	0.009	0.027	81.40	1.55	1.32
Diclofenac	0.9997	y = 146,004x − 114.8	0.050	0.151	86.25	2.89	2.63
Primidone	0.9968	y = 28,705x + 1206.3	0.709	2.127	86.10	2.61	3.12

**Table 5:** Pharmaceuticals with increased concentrations from both patients and the healthy group.

Pharmaceuticals (µg/L)	Hospital Patients		Healthy Individuals	
	Before Storage (µg/L)	After Storage (µg/L)	Before Storage (µg/L)	After Storage (µg/L)
Lamivudine	<LOD	-	ND	116.92
Acetaminophen	<LOD	-	ND	62.28
Diclofenac	<LOD	-	ND	35.81
Caffeine	61.89	155.74	ND	ND
Sulfamethoxazole	<LOD	-	ND	12.71
Sulf-hydroxylamine	ND	40.33	ND	22.0
Nevirapine	10.80	85.86	<LOD	
Prednisolone	27.34	48.91	24.08	52.21
17α-ethinylestradiol	14.73	44.58	<LOD	
Rifampicin	7.82	11.47	17.73	17.98

## Data Availability

The original contributions presented in this study are included in this article. Further inquiries can be directed to the corresponding authors.
